# Comprehensive analysis of lncRNAs involved in skeletal muscle development in ZBED6-knockout Bama pigs

**DOI:** 10.1186/s12864-021-07881-y

**Published:** 2021-08-04

**Authors:** Dandan Wang, Yabin Pu, Yefang Li, Dengke Pan, Shengnan Wang, Wenjie Tian, Yuehui Ma, Lin Jiang

**Affiliations:** 1 Laboratory of Animal (Poultry) Genetics Breeding and Reproduction, Ministry of Agriculture, Institute of Animal Sciences, Chinese Academy of Agricultural Sciences (CAAS), Beijing, P. R. China; 2 National Germplasm Center of Domestic Animal Resources, Ministry of Technology, Institute of Animal Sciences, Chinese Academy of Agricultural Sciences (CAAS), Beijing, P.R. China; 3 Clinical Immunology Translational Medicine Key Laboratory of Sichuan Province, Sichuan Academy of Medical Sciences & Sichuan Provincial People’s Hospital, 610072 Chengdu, China; 4 State Key Laboratory for Conservation and Utilization of Subtropical Agro-bioresources, College of Animal Science and Technology, Guangxi University, Nanning, P.R. China

**Keywords:** ZBED6 KO pig, lncRNAs, RNA-Seq, Skeletal muscle development

## Abstract

**Background:**

The mutation of insulin-like growth factor 2 (IGF2 mutation) that a single-nucleotide substitution (G→A) in the third intron of *IGF2* abrogates the interaction with zinc finger BED-type containing 6 (ZBED6) and leads to increased muscle mass in pigs. IGF2 mutation knock-in (IGF2 KI) and ZBED6 knockout (ZBED6 KO) lead to changes in *IGF2* expression and increase muscle mass in mice and pigs. Long noncoding RNAs (lncRNAs) may participate in numerous biological processes, including skeletal muscle development. However, the role of the ZBED6-lncRNA axis in skeletal muscle development is poorly characterized.

**Results:**

In this study, we assembled transcriptomes using RNA-seq data published in previous studies by our group and identified 11,408 known lncRNAs and 2269 potential lncRNAs in seven tissues, heart, longissimus dorsi, gastrocnemius muscle, liver, spleen, lung and kidney, of ZBED6 KO (lean mass model) and WT Bama pigs. ZBED6 affected the expression of 1570 lncRNAs (differentially expressed lncRNAs [DE-lncRNAs]; log2-fold change ≥ 1, nominal *p*-value ≤ 0.05) in the seven examined tissues. The expressed lncRNAs (FPKM > 0.1) exhibited tissue-specific patterns in WT pigs. Specifically, 3410 lncRNAs were expressed exclusively in only one tissue. Potential functions of lncRNAs were indirectly predicted by searching their target cis- and trans-regulated protein-coding genes. LncRNAs with tissue-specific expression influence numerous genes related to tissue functions. Weighted gene coexpression network analysis (WGCNA) of 1570 DE-lncRNAs between WT and ZBED6 KO pigs was used to define the following six lncRNA modules specific to different tissues: skeletal muscle, heart, lung, spleen, kidney and liver modules. Furthermore, by conjoint analysis of longissimus dorsi data (tissue-specific expression, muscle module and DE-lncRNAs) and ChIP-PCR revealed *NONSUSG002145.1* (adjusted *p*-values = 0.044), which is coexpressed with the *IGF2* gene and binding with ZBED6, may play important roles in ZBED6 KO pig skeletal muscle development.

**Conclusions:**

These findings indicate that the identified lncRNAs may play essential roles in tissue function and regulate the mechanism of ZBED6 action in skeletal muscle development in pigs. To our knowledge, this is the first study describing lncRNAs in ZBED6 KO pigs. These results may open new research directions leading to a better understanding of the global functions of ZBED6 and of lncRNA functions in skeletal muscle development in pigs.

**Supplementary Information:**

The online version contains supplementary material available at 10.1186/s12864-021-07881-y.

## Background

With the rapid development of RNA sequencing (RNA-seq) technologies, tens of thousands of noncoding RNAs have been discovered [1, 2]. Long noncoding RNAs (lncRNAs) are defined as autonomously transcribed noncoding RNAs greater than 200 nt in length that do not overlap with annotated coding genes [3]. Previously, lncRNAs, which have lower expression and protein-coding potential than mRNAs, had long been regarded as transcription junk [4]. However, in recent decades, increasing evidence has indicated that lncRNAs play important roles in many biological processes, such as the regulation of skeletal muscle development [5, 6], cell fate decisions [7], and subcutaneous fat deposition [8].

Zinc finger BED domain containing protein 6 (ZBED6), which regulates skeletal muscle development through insulin-like growth factor 2 (*IGF2*), is a transcriptional repressor [9–12]. A single nucleotide transition from G to A in intron 3 of *IGF2*, a paternally expressed quantitative trait locus (QTL) in pigs, abrogates ZBED6-IGF2 binding and results in 3-fold greater postnatal expression of *IGF2* mRNA in skeletal muscle, leading to increased muscle mass and heart size and reduced fat deposition in pigs. Recently, reports showed that ZBED6 regulates *IGF2* mRNA expression in several human and murine cell lines and in mice [13–17]. Pigs are among the most essential commercially farmed animals and have attracted substantial attention in the context of muscle development. With long-term selection for lean meat content, the QTL in *IGF2* is fixed in modern commercial pigs, such as Large White, Landrace and Hampshire [11], but most indigenous Chinese breeds homozygous for the *IGF2* wild-type allele have a lower rate of lean meat [18]. IGF2 knock-in (IGF2 KI) and ZBED6 knockout (ZBED6 KO) leads to increased IGF2 expression and muscle mass in mice and pigs [19–22]. The functional role of ZBED6, in addition to its important role in regulating *IGF2* expression, remains unclear. Hence, it is essential to elucidate the function of ZBED6 in pigs.

Recently, we obtained ZBED6 KO pigs that showed more muscle mass than wild-type (WT) pigs and analysed the effect of ZBED6 on gene expression [22], but the relationship of ZBED6 and lncRNAs was unclear. The ZBED6 KO pig provides a good lean mass pig model to illustrate expression profiles and the functions of lncRNAs in skeletal muscle development. Here, we performed RNA-seq analysis on seven different tissues of ZBED6 KO and WT pigs to systematically identify lncRNAs. Differentially expressed lncRNAs (DE-lncRNAs), tissue expression patterns and potential functions were elucidated. Our study will be of great use in future explorations of skeletal muscle lncRNA and ZBED6 functions.

## Results

### Identification and characteristics of lncRNAs expressed in different tissues of WT and ZBED6 KO pigs

**Fig. 1:**
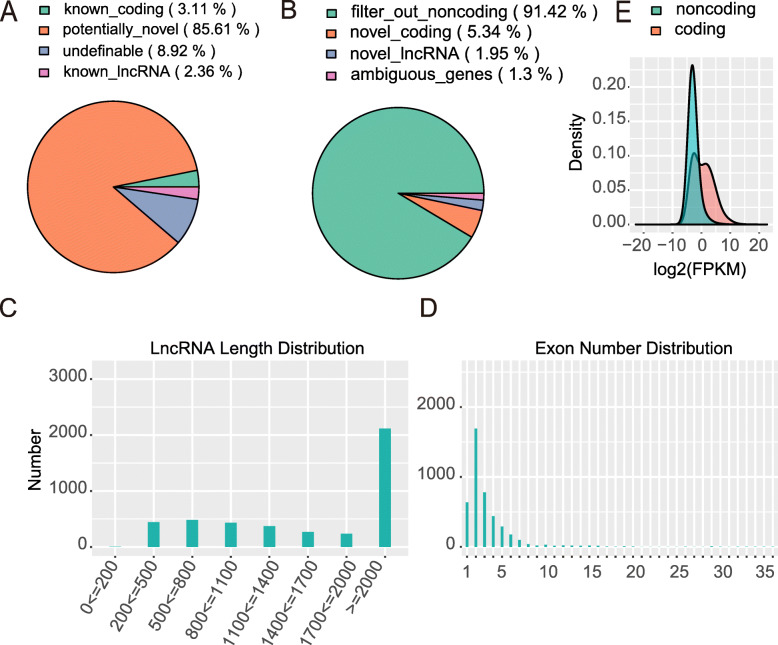
Statistics of lncRNAs identified in this study. (**A**) Percentage of known lncRNA transcripts. (**B**) Percentage of potentially novel transcripts. (**C**) Length distribution of lncRNAs. (**D**) Exon number of lncRNAs. (**E**) Expression levels of mRNAs and lncRNAs

To comprehensively investigate the lncRNAs in WT and ZBED6 KO pigs, the Illumina HiSeq 2500 platform was used to obtain a comprehensive view of lncRNAs in seven tissues, namely, muscle (gastrocnemius muscle and longissimus dorsi), heart, liver, spleen, lung and kidney, between ZBED6 KO and WT pigs, with three biological replicates for each group (a total of 42 samples). We generated approximately 4.2 to 8.74 G of raw reads, and 70 %~87.3 % of the clean reads mapped to the genome of the paired reads for each sample (Supplementary Table 2). The GC content of the clean data ranged from 47.05 to 57.92 %, demonstrating that the reliability and quality of the sequencing data were adequate for further analysis. To identify lncRNAs, the transcripts were assembled and reconstructed into a total of 210,858 transcripts using Cufflinks software. Ultimately, in addition to 11,408 known lncRNAs, 4352 potential lncRNA transcripts that overlapped 2269 lncRNA genes were identified by using CNCI software (Fig. 1 A-B). It is important to maximize our understanding of lncRNAs through multiple structural features. We found that 48.59 % of the lncRNAs were more than 2000 bp (Fig. 1 C), whereas 85.39 % spanned two or more exons (Fig. 1D). LncRNAs tended to have lower expression levels than mRNAs (Fig. 1E). The features of lncRNAs were consistent with those of lncRNAs reported in other studies.

**Fig. 2:**
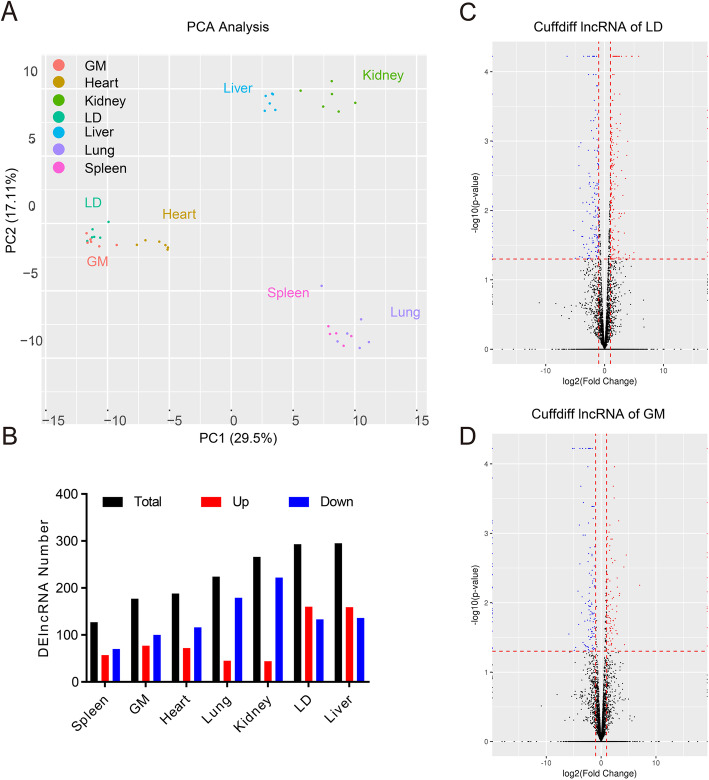
Differentially expressed lncRNAs. (**A**) PCA of the expressed lncRNAs in multiple tissues in WT and ZBED6 ^−/−^ pigs (*n* = 3). (**B**) The number of DE-lncRNAs in each tissue. (**C**-**D**) Volcano plot showing 293 and 177 DE-lncRNAs of longissimus dorsi (LD) and gastrocnemius muscle (GM) between WT and ZBED6^−/−^ pigs (*n* = 3)

Principal component analysis (PCA) of these 42 samples clearly showed that the lncRNA expression profiles of seven tissues were clustered together separately, and the profiles were similar in skeletal muscle (gastrocnemius muscle and longissimus dorsi) (Fig. 2 A). We detected 13,677 expressed lncRNAs in this experiment Using log2-fold chang ≥ 1, nominal *p*-value ≤ 0.05 as the filter criteria,we found 127 to 295 DE-lncRNAs across seven tissues between the WT and ZBED6 KO samples (Fig. 2B), of which 177 and 293 DE-lncRNAs were identified in gastrocnemius muscle and longissimus dorsi, respectively (Fig. 2 C-D). Using log2-fold change ≥ 1, adjusted *p*-values ≤ 0.05 as the filter criteria, 82 to 211 DE-lncRNAs were detected, of which 116 and 211 DE-lncRNAs were found in gastrocnemius muscle and longissimus dorsi, respectively (Supplementary Table 3). The details of DE-lncRNAs in the seven tissues are shown in Supplementary Table 3. These results suggest that ZBED6 regulates the expression of lncRNAs in pigs.

### Tissue-specific expression of lncRNAs in WT pig

**Fig. 3:**
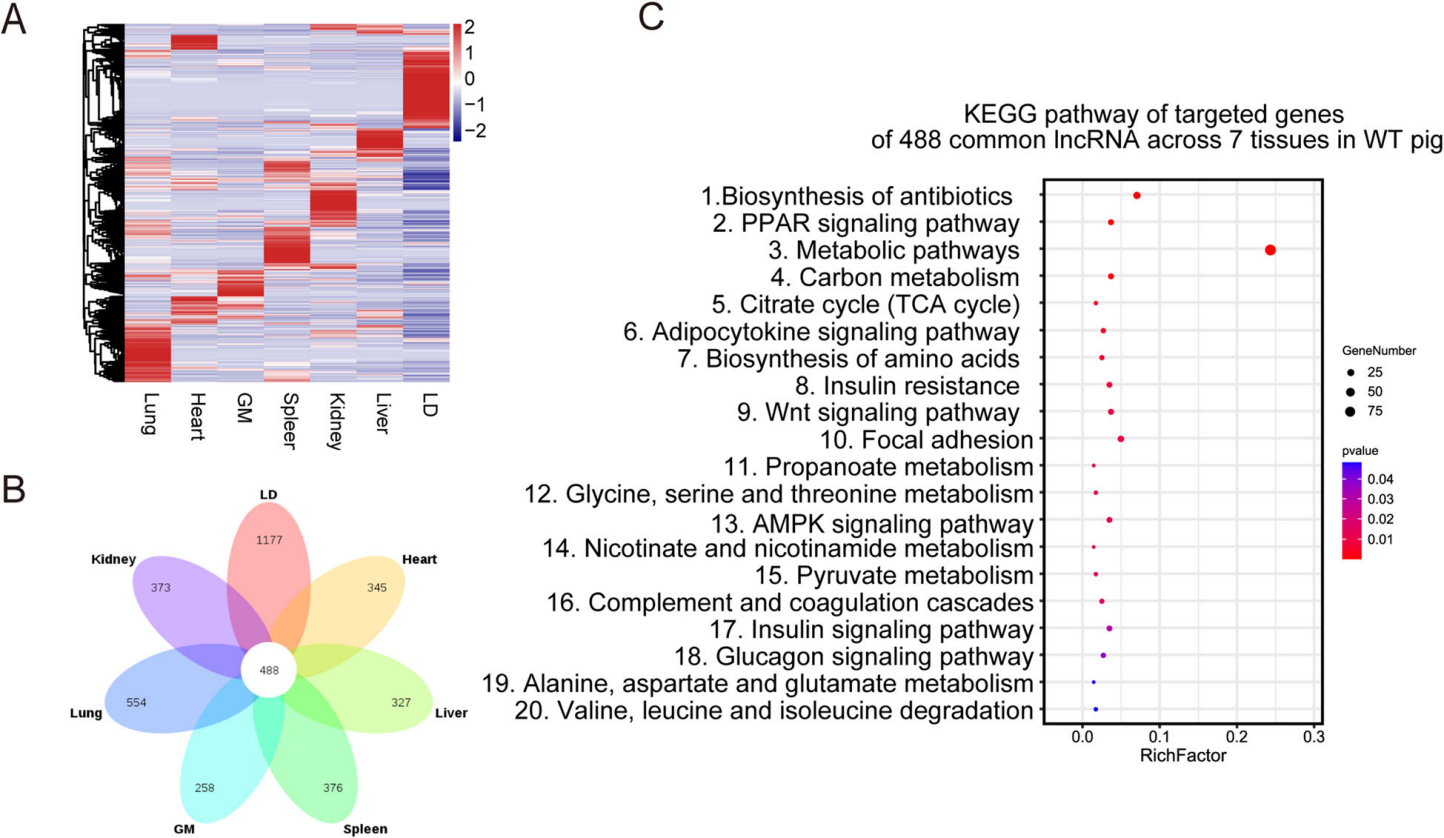
Tissue-specific expression of lncRNAs in WT pig. (**A**) Heatmap of expressed lncRNAs across 7 tissues. Each row represents the expression levels of all detected lncRNAs, and each column contains all expressed transcripts. We transformed the FPKM values into log2(FPKM + 1) values and then calculated the Z-score for every log 2 (FPKM + 1) value within each tissue. (**B**) Venn diagram of expressed lncRNAs in seven tissues. (**C**) Enriched gene pathway terms of targeted genes of 488 commonly expressed lncRNAs across 7 tissues [23]

To elucidate the functional roles of lncRNAs in WT pigs, we used lncRNA expression data (FPKM > 0.1) from the seven different tissues from three WT pigs to characterize tissue-specific expression patterns. A heatmap based on the expression patterns in all tissues was generated across all expressed lncRNAs, which indicated that the majority exhibited a preferential tissue expression pattern (Fig. 3 A). A total of 488 commonly expressed lncRNAs were found in all tissues, and 3410 lncRNAs displayed exclusive expression in one tissue (Supplementary Table 4), with the most tissue-specific lncRNAs present in the longissimus dorsi (*N* = 1177) and the fewest tissue-specific lncRNAs present in the gastrocnemius muscle (N = 258) (Fig. 3B). To analyse the possible roles of these lncRNAs in pig tissues, potential cis- and trans-target genes for lncRNAs were predicted. As a result, a total of 1815 cis- and 1804 trans-target genes for common and tissue-specific expressed lncRNAs were detected (Supplementary Table 5). Target genes of the 488 commonly expressed lncRNAs, including adiponectin (*ADIPOQ*), peroxisomal proliferating-activated receptor gamma (*PPARG*), anabolic-androgenic steroids (*AASS*) and *WNT9B*, play central roles in the regulation of growth and fat development in pigs and were enriched in 20 KEGG pathways, including biosynthesis of antibiotics, the peroxisome proliferator-activated receptor (*PPAR*) signalling pathway and the Wnt signalling pathway (Fig. 3 C). Additionally, tissue-specific lncRNAs of the seven tissues were enriched in metabolic pathways, the intestinal immune network for IgA production, fatty acid metabolism and transcriptional misregulation in cancer (Supplementary Table 6).

### Tissue expression patterns of lncRNAs in WT and ZBED6 KO pigs

**Fig. 4:**
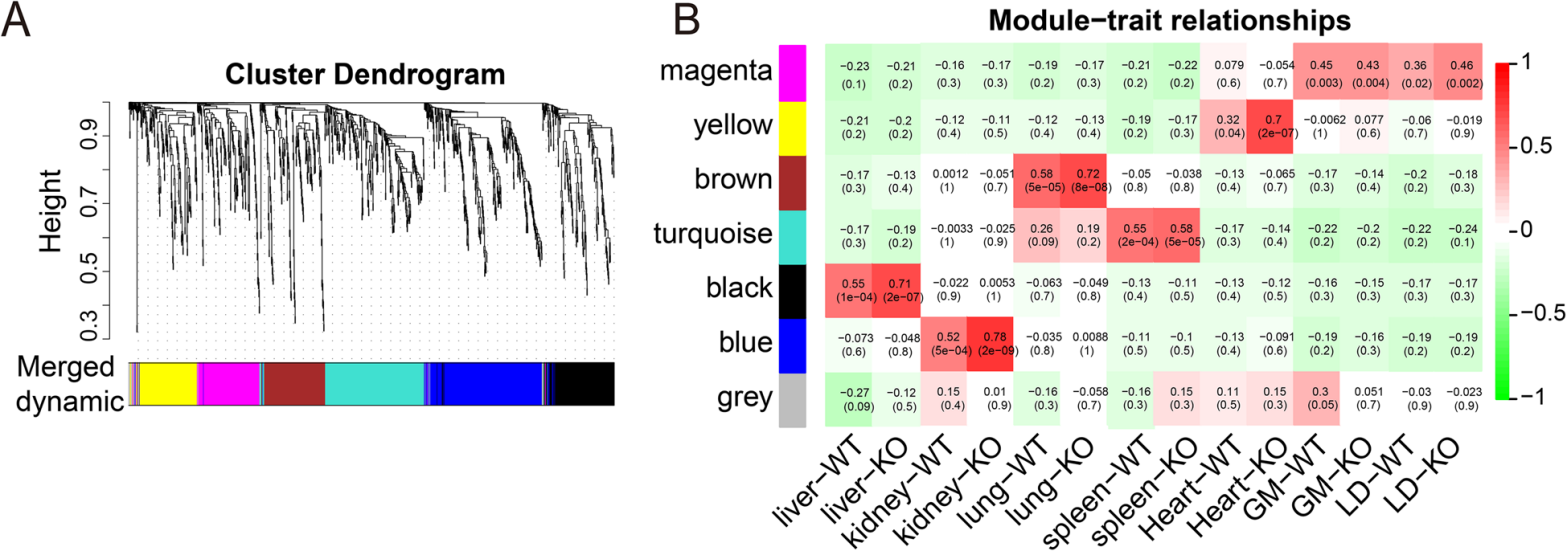
Expression modules of lncRNAs in WT and ZBED6 KO pigs determined by WGCNA. (**A**) Hierarchical cluster tree of all differentially expressed lncRNA modules. Modules correspond to the branch and are marked by a colour, as indicated by colour strips under the tree. (**B**) Correlation analysis between lncRNA modules and tissues

To demonstrate the dynamics of lncRNA expression in different tissues of WT and ZBED6 KO pigs, the expression patterns of DE-lncRNAs (nominal *p*-value ≤ 0.05) were clustered using weighted gene coexpression network analysis (WGCNA). A total of 7 lncRNA transcriptional modules were identified in WT and ZBED6 KO pig tissues, with six modules showing a strong correlation with specific tissues (Fig. 4 A-B). We focused on modules that were highly positively correlated with tissues (correlation > 0.3, *p*-value < 0.05) and defined six tissue-specific modules (Supplementary Table 7). The magenta module, which included 163 lncRNAs strongly related to the four muscle tissues (gastrocnemius muscle and longissimus dorsi of WT and ZBED6 KO pigs), was identified as a skeletal muscle module, whereas grey indicated weak correlation with all tissues (Fig. 4B). The yellow, brown, turquoise, blue and black modules showed continuously positive correlations with heart, lung, spleen, kidney and liver tissues in WT and ZBED6 KO pigs, respectively.

### LncRNAs play a potential role in skeletal muscle development in ZBED6 KO pigs

**Fig. 5:**
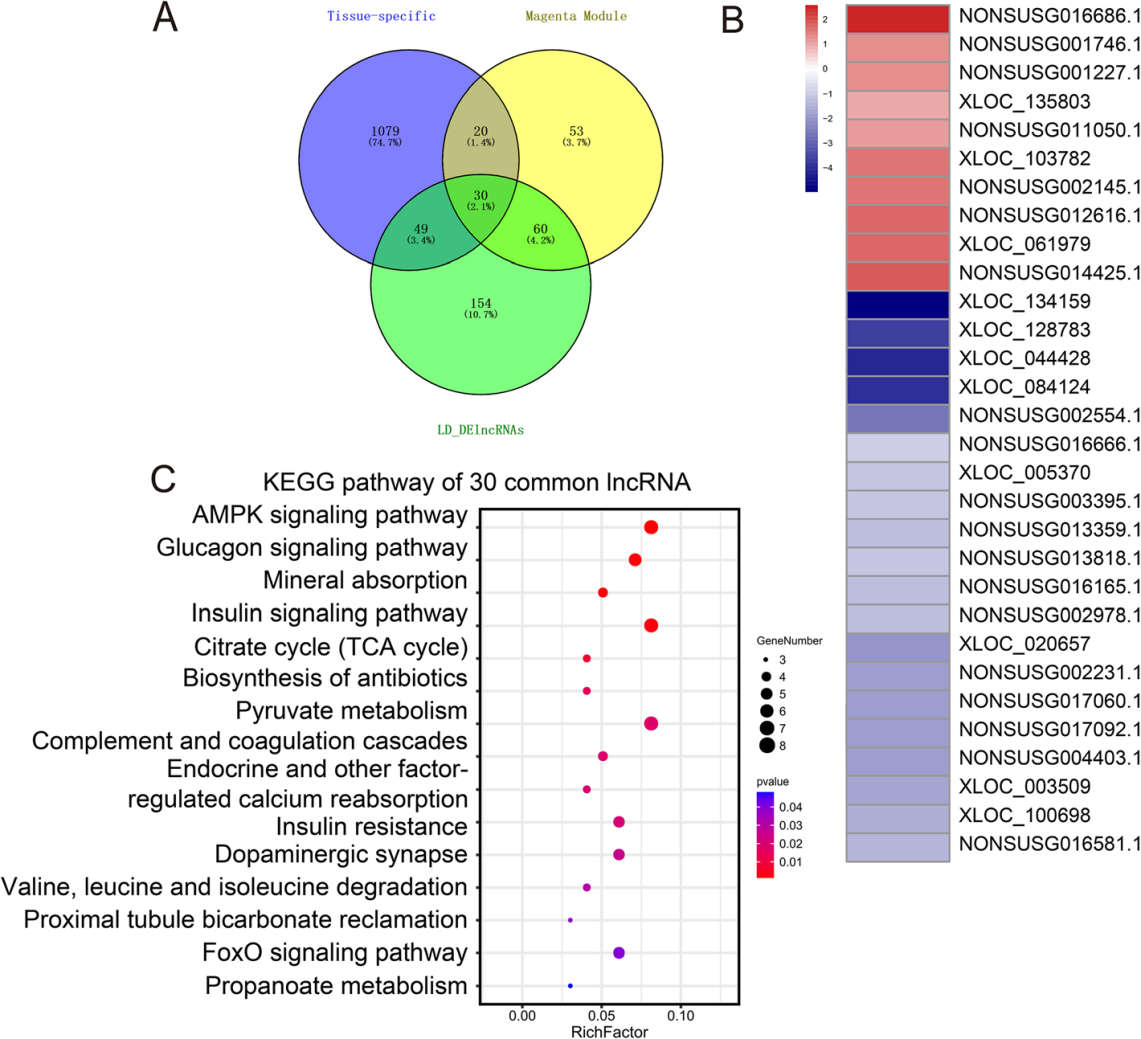
LncRNAs in longissimus dorsi (LD) development in ZBED6 KO pigs. (**A**) Thirty common lncRNAs in longissimus dorsi tissue-specific expression (tissue-specific), the skeletal muscle module (magenta module) and DE-lncRNAs of longissimus dorsi (LD_DE-lncRNAs). (**B**) Heatmap with log2(fold-change) between WT and ZBED6 KO pig longissimus dorsi of 30 common lncRNAs. (**C**) Enriched gene pathway terms of 30 common lncRNA-targeted genes [23]

**Fig. 6:**
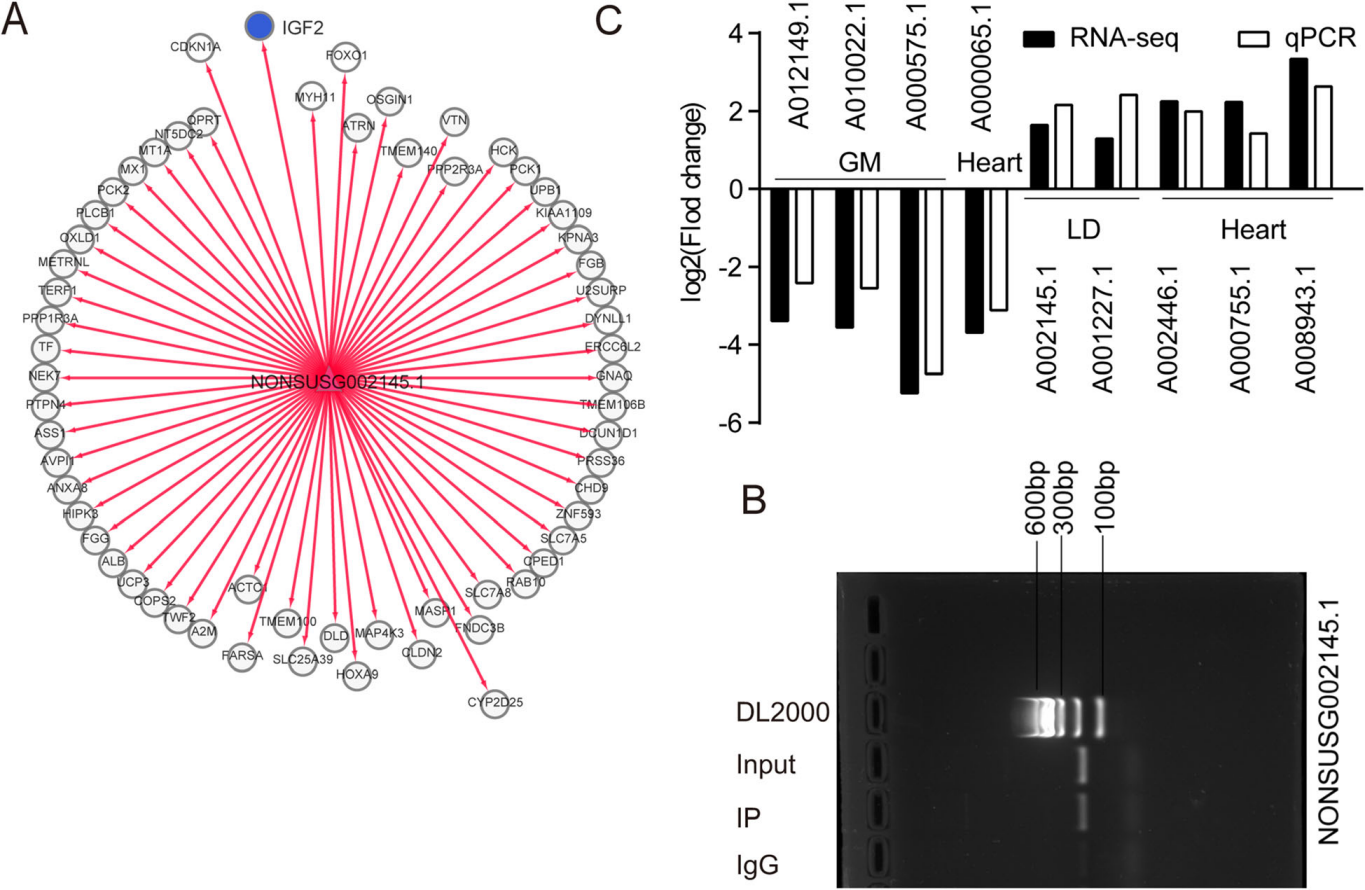
Functional network of the key lncRNA in longissimus dorsi (LD) development in ZBED6 KO pigs. (**A**) Function network of NONSUSG002145.1. (**B**) ChIP-PCR analysis in longissimus dorsi of ZBED6 occupancy at the binding sites of NONSUSG002145.1. The red triangle represents lncRNA, the circle represents target mRNA of lncRNA, the blue circle represents target mRNA IGF2 related to muscle development. (**C**) qRT-PCR validation

To identify the function of lncRNAs in skeletal muscle development in ZBED6 KO pigs, we overlapped the lncRNAs from three analyses: longissimus dorsi tissue-specific expression, skeletal muscle module (magenta module) and DE-lncRNAs (nominal *p*-value ≤ 0.05) of the longissimus dorsi. Thirty common lncRNAs were revealed (Fig. 5 A), of which eleven lncRNAs were novel and nineteen lncRNAs were known. However, no common lncRNAs of gastrocnemius muscle were revealed with a similar analysis. Therefore, the study mostly was focused on longissimus dorsi. Heatmap analysis further showed that the expression of ten of the thirty common lncRNAs was upregulated in the longissimus dorsi of ZBED6 KO pigs (Fig. 5B). These results suggested that lncRNAs play an important role in skeletal muscle development in ZBED6 KO pigs. To explore how lncRNAs work in concert with their target genes (mRNAs) to regulate skeletal muscle development and to identify the key molecules, possible regulatory networks of interactions between lncRNAs and their target genes (mRNAs) were constructed. In the present study, we predicted potential cis- and trans-target genes of DE-lncRNAs and further constructed a lncRNA-gene interaction network between the DE-lncRNAs and their corresponding differentially expressed cis- and trans-target genes by using Cytoscape. For the thirty common lncRNAs in longissimus dorsi, we detected 21 cis and 268 trans target genes (Supplementary Table 8) and performed KEGG enrichment analysis to reveal the overrepresented biological processes of the 289 target genes using DAVID (Fig. 5 C). The KEGG results showed that the genes were related to the myocyte proliferation or differentiation pathway, the AMPK signalling pathway, the FoxO signalling pathway, factor-regulated calcium reabsorption and others (Supplementary Table 9). In addition, metabolic pathways, the citrate cycle (TCA cycle), glucagon signalling pathway and pyruvate metabolism were overrepresented. The lncRNA-mRNA and ChIP-PCR analysis showed that among the thirty common lncRNAs, *NONSUSG002145.1* that was upregulated in the longissimus dorsi of ZBED6 KO pigs (adjusted *p*-values = 0.044), is the target of ZBED6 and perfectly coexpressed with a crucial regulator of skeletal muscle development, IGF2 (Fig. 6 A-B), suggesting that *NONSUSG002145.1* could be potential regulators of IGF2 expression during skeletal muscle development.

### RT-qPCR Validation of DE-lncRNAs

To validate the RNA sequencing data, 9 lncRNAs were randomly selected from DE-lncRNAs, and their expression levels in different tissues of WT and ZBED6 KO pigs were examined by RT-qPCR. The results showed that the expression of the 9 lncRNAs was consistent with the expression trends calculated from the RNA-seq data (Fig. 6 C).

## Discussion

Thousands of lncRNAs have been identified and implicated in reprogramming [24], embryonic development [25, 26], imprinting contro l[27], cell differentiation and development [28, 29], and skeletal muscle growth [30, 31] using RNA-seq analyses, suggesting that RNA-seq is highly effective for the discovery of lincRNAs. Studies targeting lncRNAs in humans and mice have become a research hotspot in recent years. There are many excellent reviews with a specific focus on these functional and regulatory issues [3, 28, 32–34]. Compared to research on lncRNAs in humans and mice, studies on lncRNAs are still in their infancy in pigs.

ZBED6 is a transcriptional repressor and involved in skeletal muscle growth by regulating *IGF2* expression [9–16]. Recently, pigs and mice with genetically modified IGF2 and ZBED6 showed increased muscle mass and *IGF2* expression [17, 20–22]. The functional role of ZBED6 in relation to lncRNAs is still poorly characterized. Therefore, we obtained a lean mass model pig by knocking out ZBED6 and generated 545 G of RNA-seq data, which were made publicly available [22]. Using RNA-seq data we identified more than 13,000 lncRNAs in seven tissues. These lncRNAs showed a shorter length, fewer exons and lower expression levels than mRNAs, which is consistent with findings in humans [35], mice [36], sheep [29, 30] and horses [37]. Almost 83.41 % of the identified lncRNAs (11,408) in our study were previously reported in a pig RNA-seq study [5]. The identification of potentially novel lncRNAs might be due to differences in various aspects of these studies, such as types of pigs and sampled tissue. In addition, the RNA-seq analysis and RT-qPCR validation indicated that the lncRNAs are bona fide transcripts rather than random products from transcriptional noise.

Several studies have already revealed that lncRNAs tend to show tissue-specific expression [38, 39]. Studies in humans [35] and sheep [29] have shown that tissue-specific lncRNAs are involved in certain biological processes in specific tissues. Target gene analysis of lncRNAs indicated high correlation between lncRNAs and mRNAs, consistent with previous findings showing that lncRNAs were likely to be functionally associated with their nearest neighbouring mRNAs [40]. That is, the functions of lncRNAs can be predicted through their target mRNAs. We found 488 commonly expressed lncRNAs across the seven tissues in WT pigs, including 1213 lncRNA-mRNA pairs that regulate growth and fat development. *ADIPOQ* and *PPARG*, which are involved in fat metabolism pathways (PPAR signalling pathway and adipocytokine signalling pathway), play a crucial role in adipogenic induction [19, 41]. *WNT9B*, a secreted Wnt growth factor enriched in the development and growth pathway (Wnt signalling pathway), is a developmental regulator [42]. This suggested that lncRNAs have various roles in many biological processes in pigs, such as muscle development, metabolism and immunity, consistent with previous studies [26, 43–46]. These data provide a high-quality lncRNA resource for pigs.

293 DE-lncRNAs (nominal *p*-value ≤ 0.05) were found in the muscles of WT and ZBED6 KO pigs, of which the adjusted *p*-values of 211 DE-lncRNAs was less than 0.05. And 163 specific lncRNAs were observed in the muscles of WT and ZBED6 KO pigs, suggesting that ZBED6 is associated with the growth of pig tissues by regulating lncRNAs. Interestingly, we identified *NONSUSG002145.1* (adjusted *p*-values = 0.044) which passed multiple testing or FDR correction, coexpressed with *IGF2*, could bind with ZBED6 directly. Due to the central role of *IGF2* in skeletal muscle development in mice and pigs [9, 10, 47], *NONSUSG002145.1* that was reported rarely may have regulatory roles in the expression of *IGF2* during the development of muscle in ZBED6 KO pigs. Therefore, additional studies, such as loss-of-function experiments, are needed in the future to provide further insights into the regulatory functions of *NONSUSG002145.1* in skeletal muscle development.

## Conclusions

In the current study, we identified and characterized lncRNAs in WT pigs. LncRNAs with tissue-specific expression across seven tissues in WT pigs were discovered. Some lncRNAs expressed across all tissues tested may influence the expression of numerous genes, including those involved in (1) fat metabolism factors (e.g., *ADIPOQ* and *PPARG*) and (2) development and growth factors (e.g., *WNT9B*). We also examined the effects of ZBED6 on the lncRNA expression profile and identified DE-lncRNAs between WT and ZBED6 KO pigs. WGCNA revealed lncRNA tissue expression patterns in WT and ZBED6 KO pigs. *NONSUSG002145.1* (adjusted *p*-values = 0.044), regulating the expression of *IGF2*, were identified as key molecules related to muscle development in ZBED6 KO pigs.

## Methods

### Ethics statement

All animal experiments were performed according to protocols and guidelines approved by the Institutional Animal Care and Use Committee of the Beijing Academy of Agricultural Sciences.

### Datasets

All RNA-seq data of female ZBED6 KO and female WT Bama miniature pigs used in this study were from previous studies in our laboratory that have been deposited in the Sequence Read Archive (https://www.ncbi.nlm.nih.gov) with the indicated accession codes (BioProject ID: PRJNA663759). The RNA-seq data included seven tissues (heart, longissimus dorsi, gastrocnemius muscle, liver, spleen, lung and kidney) from three ZBED6 KO and three WT Bama pigs (Supplementary Table 10).

### Sequence analysis

Raw reads were cleaned by removing adapter sequences, reads with over 10 % N sequences, and low-quality reads in which the number of bases with a quality value Q ≤ 10 was more than 50 %, and then clean reads were obtained. The Q20, Q30, and GC contents of the clean data were calculated. The clean reads were mapped to the pig reference genome (https://www.ncbi.nlm.nih.gov/genome/84?genome_assembly_id=317145) using TopHat (v2.0.11) with default parameters [48]. The BAM files generated from TopHat were assembled, and transcripts were constructed by Cufflinks (v2.2.1) [48]. Then, lncRNA analyses were performed using the lncRNA calling protocol and CNCI [49, 50]. RNA length ≥ 200 nt, exon number = > 2, CNCI score = > 0 were used to evaluate the coding potential of transcripts. We applied Cuffdiff (part of Cufflinks) to detect DElncRNAs between the ZBED6 KO and WT pig. Using the negative binomial distribution, *p*-value was calculated to estimate changes in a transcript’s fragment count. Then *p*-value was corrected multiply to calculate *q*-value (adjusted *p*-values) by false discovery rate (FDR) and the Storey’s q-value procedure [51]. Transcripts with fold changes ≥ 2.0 and nominal *p*-value ≤ 0.05 were identified as DElncRNAs. Cufflinks and Cuffdiff were used as described by Trapnell et al [48].

### PCA and functional enrichment analysis

The FPKM values for all of the annotated transcripts from the seven tissue transcriptomes were used to perform PCA, which was implemented by gmodels in R (version 3.1.3, http://cran.r-project.org/). GO enrichment analysis of differentially expressed genes was implemented using DAVID (https://david.ncifcrf.gov/). Pig genes were used as the background gene set when performing the KEGG enrichment analysis [23]. P values ≤ 0.05 were considered significant.

### Tissue-specific identification of lncRNAs

We used RNA-seq data of seven tissues (heart, longissimus dorsi, gastrocnemius muscle, liver, spleen, lung and kidney) from WT pigs to characterize the expression patterns of the lncRNA genes. The heatmap was generated using all expressed lncRNA genes in seven tissues using the pheatmap package in R based on the FPKM values (FPKM > 0.1). The level of gene expression is visualized by using a colour gradient from blue to red. For seven tissues from WT pigs, lncRNAs were classified into tissue-specific lncRNAs that were observed in only one tissue.

### WGCNA Network Analysis

The genes that were differentially expressed between all stages of seven tissues from WT and KO pigs were selected to identify key genes in tissue development using the R package WGCNA [52]. A signed weighted correlation network was constructed by first creating a matrix of Pearson correlation coefficients between all pairs of genes across the measured samples. The adjacency matrix was then transformed into a topological overlap matrix (TOM) to minimize the effects of noise and spurious associations. To define modules as branches, we employed the dynamic tree cut algorithm with default parameters to cut the hierarchal clustering tree [53].

### Target Gene Prediction

A previous study showed that lncRNAs exert cis-regulatory effects on their colocalized genes [54]. The colocalization role is lncRNA acting on neighbouring target genes. The 10 k coding genes upstream and downstream of lncRNAs were searched to identify their cis-acting effects. Additionally, lncRNAs recognize targeted genes at the expression level. The Pearson correlation coefficient method was used to analyse the correlation between lncRNAs and mRNAs. mRNAs with an absolute correlation value greater than 0.9 and a *p*-value less than 0.05 were identified as trans target genes.

### Chromatin immunoprecipitation (ChIP)-PCR analysis

ChIP was conducted previously described [55]. Briefly, freshly longissimus dorsi were treated with 1 % formaldehyde for 18 min and neutralizing with glycine (AMRESCO, USA) for 5 min at room temperature. Then longissimus dorsi was smashed and resuspended in SDS lysis buffer (Beyotime, China). After incubation for 20 min at 4 °C, the lysates were sonicated 24 times (30 s each) (Bioruptor® Sonication System, USA). Total solution and Protein G agarose beads (ThermoFisher Scientific, USA) were incubated at 4℃ overnight with 10ug of the following antibodies: anti-ZBED6 antibody (HPA068807, ATLAS) and normal mouse IgG antibody (2729 S, CST). The beads were washed to get ChIP Elution Buffer. Add 20 µl 5 M NaCl to the combined eluates and reverse histone-DNA crosslinks by heating at °65 for 4 h. RNase A (TIANGEN, China) and Proteinase K (TIANGEN, China) were added to ChIP Elution Buffer to remove RNA and proteins. ChIP DNA Clean &Concentrator purification spin column (ZYMO, Irvine, California, USA) was used to purify coprecipitated DNA. The ZBED6 binding site, *NONSUSG002145.1* was evaluated using PCR and normalized by total chromatin (input). Normal mouse IgG was used as the negative control, the primers are described in supplementary Table 1. The PCR conditions were: 1 cycle at 95℃ for 5 min followed by 33 cycles at 95℃ for 30 s, 58–59℃ for 30 s, and 72℃ for 30 s. The PCR products were then electrophoresed on 1.5 % agarose gels stained with GelGreen (TIANGEN, China).

### Quantitative PCR

For the qRT-PCR analysis of genes, single-strand cDNA was synthesized from RNA using the PrimeScript RT reagent Kit With gDNA Eraser (Takara, China). The qPCR reactions was performed in ABI MicroAmp Optical 96-well Reaction plates on an ABI 7500 real-time PCR instrument (USA), using SYBR Premix Ex Taq (Tli RNaseH Plus; Takara). The primer sequences, designed using Premier Primer 5.0 software, are listed in Supplementary Table 1. Relative gene expression was calculated using the comparative cycle threshold (2^−ΔΔCt^) method.

### Statistical analysis

Statistical analyses of the qRT-PCR results and graphs were carried out in GraphPad Prism 7 (GraphPad Software Inc, La Jolla, CA, USA). Statistical significance of the data was tested by performing paired t-tests. The P values were determined by Student’s t test (unpaired). Error bars indicate SEM. ***, *P* < 0.001; **, *P* < 0.01; *, *P* < 0.05.

## Supplementary Information

**Additional file 1.** **Supplementary Table S1.** Primer pairs of DEGs used for qRT-PCR validation.

**Additional file 2.** **Supplementary Table S2.** Quality analysis and genome mapping analysis of transcriptome sequencing.

**Additional file 3.** **Supplementary Table S3.** List of DE-lncRNAs in 7 tissues (gastrocnemius muscle, longissimus dorsi, heart, liver, spleen, lung and kidney) between WT and ZBED6 KO pigs.

**Additional file 4.** **Supplementary Table S4.** Tissue-specific and common expression of lncRNAs across 7 tissues (gastrocnemius muscle, longissimus dorsi, heart, liver, spleen, lung and kidney) in WT pigs.

**Additional file 5.** **Supplementary Table S5.** Cis and trans target genes predicted to be regulated by tissue-specific and commonly expressed lncRNAs in 7 tissues (gastrocnemius muscle, longissimus dorsi, heart, liver, spleen, lung and kidney) in WT pigs.

**Additional file 6.** **Supplementary Table S6.** KEGG of targeted genes of tissue-specific lncRNAs across 7 tissues in WT pigs.

**Additional file 7.** **Supplementary Table S7.** List of lncRNA modules.

**Additional file 8.** **Supplementary Table S8.** Cis- and trans-target genes predicted to be regulated by 30 commonly expressed lncRNAs in longissimus dorsi tissue-specific expression (tissue-specific), skeletal muscle module (magenta module) and DE-lncRNAs of longissimus dorsi (LD_DE-lncRNAs).

**Additional file 9.** **Supplementary Table S9.** KEGG enrichment of 30 commonly expressed lncRNAs.

**Additional file 10.** **Supplementary Table S10.** The detail of RNA-seq data.

## Data Availability

All the RNA-seq reads have been deposited in the Sequence Read Archive (https://www.ncbi.nlm.nih.gov/sra) with the accession codes (BioProject ID: PRJNA663759).

## Abbreviations

RNA-Seq: RNA sequencing
lncRNA: Long non-coding RNA
ZBED6: Zinc finger BED domain containing protein 6
IGF2: Insulin-like growth factor 2
QTL: Quantitative trait locus
DE-lncRNAs: differentially expressed lncRNAs
ZBED6 KO: ZBED6 knockout
WT: Wildtype
WGCNA: Weighted gene co-expression network analysis
GM: Gastrocnemius muscle
LD: Longissimus dorsi
TOM: Topological overlap matrix
PCA: Principal component analysis
ADIPOQ: Adiponectin
PPARG: Peroxisomal proliferating-activated receptor gamma
AASS: Anabolic-androgenic steroids
PPAR: Peroxisome proliferators-activated receptors
ChIP: Chromatin immunoprecipitation
